# Eyes in the wild: *Thelazia callipaeda* in wild carnivores of Croatia

**DOI:** 10.1186/s13071-025-06991-w

**Published:** 2025-08-06

**Authors:** Ema Gagović, Šimun Naletilić, Daria Jurković Žilić, Željko Mihaljević, Adnan Hodžić, Relja Beck

**Affiliations:** 1 Department of Bacteriology and Parasitology, Croatian Veterinary Institute, 10000 Zagreb, Croatia; 2 Department of Pathology, Croatian Veterinary Institute, 10000 Zagreb, Croatia; 3 Centre for Microbiology and Environmental Systems Science (CMESS), Department of Microbiology and Ecosystem Science, University of Vienna, 1010 Vienna, Austria

**Keywords:** *Thelazia callipaeda*, Wild carnivores, Seasonality, Croatia, Parasite transmission dynamics

## Abstract

**Background:**

*Thelazia callipaeda* is an emerging zoonotic nematode that infects the eyes of domestic and wild mammals across Europe and is transmitted by drosophilid flies of the genus *Phortica*. Despite increasing reports across the continent, its distribution in wildlife in southeastern Europe remains poorly understood. To our knowledge, this study represents the first comprehensive parasitological and molecular investigation of *T. callipaeda* in wild carnivores in Croatia.

**Methods:**

Between 2020 and 2023, a total of 2632 animals, either legally hunted or found dead, were examined. These included 1794 red foxes (*Vulpes vulpes*), 443 golden jackals (*Canis aureus*), 180 European badgers (*Meles meles*), and 215 stone martens (*Martes foina*). Ocular examination and nematode collection were conducted, followed by morphological identification and COI gene sequencing for species confirmation.

**Results:**

The overall prevalence of *T. callipaeda* infection was 3.9% in red foxes and 4.3% in golden jackals. No infections were detected in badgers or martens. The infections were geographically restricted to continental regions, with the highest prevalence recorded in eastern counties. Seasonal patterns were evident in red foxes, where infection rates and reproductive activity peaked in autumn and winter. In contrast, golden jackals showed more stable infection rates throughout the year. A significant proportion of infections involved gravid females, particularly in jackals (73.7%), suggesting their critical role in sustaining transmission cycles. Molecular analysis confirmed that all isolates belonged to *T. callipaeda* haplotype 1.

**Conclusions:**

These findings highlight the established presence of *T. callipaeda* in Croatian wildlife and reveal distinct host-specific patterns in prevalence, seasonality, and reproductive dynamics. Red foxes may act as seasonal reservoirs of infection, while golden jackals could serve as year-round reservoirs. Considering the zoonotic potential of this parasite, further surveillance and integrated monitoring of vectors and vertebrate hosts are essential for evaluating future risks to public and veterinary health in the region.

**Graphical abstract:**

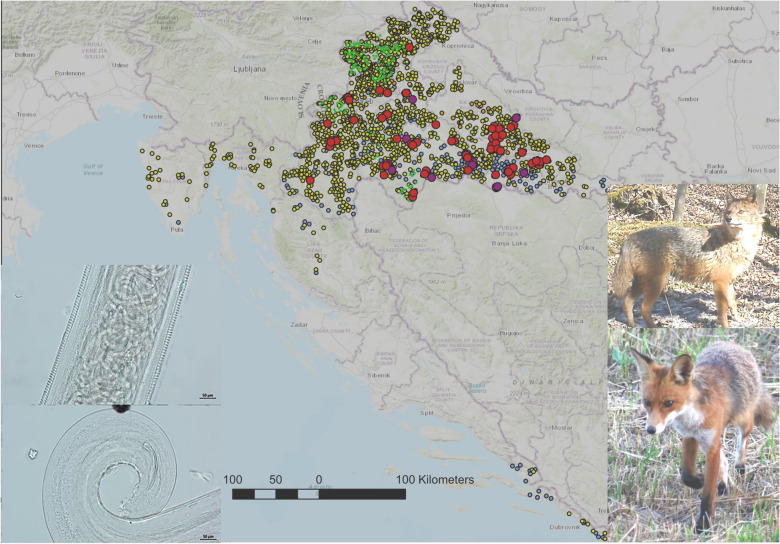

## Background

Thelaziosis is a parasitic disease of mammals caused by nematodes of the genus *Thelazia* (Spirurida, Thelaziidae). Adult worms reside in the conjunctival sac, while larval stages may also be found in the lacrimal ducts [1]. There are 16 known species of *Thelazia* that infect various mammalian hosts, with *Thelazia callipaeda* [2] being the most widespread [2]. This nematode was originally described in Asia, but it has since spread across Europe and, more recently, has been reported in North America (the US and Canada) and South America (Brazil) [3]. *Thelazia callipaeda* infects a broad spectrum of hosts, including carnivores, lagomorphs, and humans [2–4].

Infections with *T. callipaeda* can cause a variety of clinical signs depending on parasite burden and host susceptibility, ranging from mild ocular symptoms (lacrimation, conjunctivitis, and keratitis) to corneal ulceration and blindness [5]. In Europe, *T. callipaeda* has been documented in numerous domestic and wild mammals, including dogs (*Canis familiaris*), cats (*Felis catus*), red foxes (*Vulpes vulpes*), grey wolves (*Canis lupus*), stone martens (*Martes foina*), wildcats (*Felis silvestris*), golden jackals (*Canis aureus*), European badgers (*Meles meles*), brown bears (*Ursus arctos*), European hares (*Lepus europaeus*), European rabbits (*Oryctolagus cuniculus*), and humans (*Homo sapiens*) [6–11].

The life cycle of *T. callipaeda* involves indirect transmission via drosophilid flies of the genus *Phortica* [12, 13]. Although the potential for transmission by *Phortica okadai* and *P. oldenbergi* has been demonstrated under experimental conditions, *P. variegata* is currently the only species confirmed as a natural vector of *T. callipaeda* in Europe [13–15]. Within the vector, the parasite develops from the first- to the third-stage larva (L1–L3) over approximately 14–21 days. Development to the adult stage in the definitive host takes an additional 35 days. Although only male *P. variegata* feed on ocular secretions and are thus responsible for transmitting the infective L3 larvae, *T. callipaeda* larvae have also been detected in female *P. variegata* and *P. okadai* under experimental conditions [13, 16].

Since its initial identification in dogs in northern Italy in 1989 and later in red foxes between 1995 and 2002 [17, 18], *T. callipaeda* has progressively expanded its range across Europe. Reported prevalence varies considerably depending on host species and geographic region, with the highest rates observed in domestic dogs and red foxes [6, 19]. In red foxes, prevalence ranges from 1.3% in Slovakia [20] and 5.6% in Switzerland [21] to 27.7% in Bosnia and Herzegovina [22] and 29.4% in Romania [23]. In contrast, *T. callipaeda* infections in other wild carnivores such as stone martens, wildcats, wolves, badgers, and golden jackals are reported less frequently and are primarily documented in studies from Romania and Italy. Prevalence in these species is generally lower. For instance, in stone martens, prevalence ranged from 7.7% (1/13) to 13.6% (3/22); in wildcats, from 11.1% (1/9) to 37.5% (3/8); in wolves, from 7.7% (1/13) to as high as 50.0% (1/2); in badgers, 1.8% (1/55); and in golden jackals, 1.6% (1/64) [7, 8, 24].

In Croatia, *T. callipaeda* has so far been confirmed in only two dogs and one human patient, all from the eastern part of the country [22, 25]. However, no systematic studies have been conducted to assess its prevalence and distribution in wild carnivores. Therefore, this study aimed to perform a comprehensive molecular survey of *T. callipaeda* infections in red foxes, golden jackals, stone martens, and European badgers collected over 4 years across various counties in Croatia.

## Methods

### Study area

In the present study, the carcasses of 1794 red foxes, 443 golden jackals, 180 European badgers, and 215 stone martens were examined for the presence of *T. callipaeda*. Samples were collected from 15 counties across four major geographical regions of Croatia (Table 1). Northwestern Croatia included Međimurje, Varaždin, Krapina-Zagorje, Zagreb County, and the City of Zagreb. Central Croatia encompassed Bjelovar-Bilogora, Sisak-Moslavina, and Karlovac counties. Eastern Croatia (Slavonia) was represented by Požega-Slavonia and Brod-Posavina counties. The coastal and mountainous regions included Primorje-Gorski Kotar, Lika-Senj, Istria, and Dubrovnik-Neretva counties. These regions vary significantly in their topography, climate, population density, and ecological features. Northwestern and Central Croatia are characterized by lowland and hilly terrains with a temperate continental climate that includes cold winters, warm summers, and moderate annual precipitation. The landscape is a mosaic of agricultural land, river valleys, and deciduous forests, interspersed with small towns and rural settlements. Eastern Croatia, comprising part of the fertile Pannonian Basin, is predominantly flat and heavily agricultural, with vast arable areas and lower overall forest coverage. It is among the least densely populated regions of the country, allowing for more extensive natural habitats. In contrast, the coastal and mountainous counties exhibit a highly heterogeneous environment, ranging from rugged karst landscapes and forested mountains to Mediterranean coastal zones. This region combines continental, alpine, and Mediterranean climatic influences and supports rich biodiversity, including a wide range of wild vertebrate species. This broad geographical coverage enabled the assessment of parasite distribution across ecologically distinct zones, reflecting diverse environmental and climatic conditions in Croatia.

**Table 1:** Total number of examined wild carnivores by county and species

County	Red fox	Golden jackal	European badger	Beech marten
Bjelovar-Bilogora County	101	6	17	36
Požega-Slavonia County	159	2	25	21
Brod-Posavina County	35	70	0	0
Karlovac County	264	84	3	0
Sisak-Moslavina County	287	164	16	20
Primorje-Gorski Kotar County	41	1	0	0
Lika-Senj County	10	6	0	0
Istra County	20	4	0	0
Dubrovnik-Neretva County	9	16	0	0
The City of Zagreb	50	10	0	2
Međimurje County	48	1	0	0
Varaždin County	181	5	13	6
Krapina-Zagorje County	265	4	59	109
Zagreb County	324	70	47	21
Total	1794	443	180	215

The table summarizes the distribution of examined animal carcasses across 15 counties in Croatia, categorized by species: red foxes, golden jackals, European badgers, and stone martens. This broad geographic coverage provided a representative assessment of *T. callipaeda* infection across different ecological zones

### Parasitological examination

The carcasses were transported to the Laboratory for Pathology of the Croatian Veterinary Institute in Zagreb, Croatia, between March 2020 and December 2023 as part of the Croatian national rabies control campaign. All animals were legally acquired through hunting or found dead. The carcasses were examined upon arrival, with the location, sex, and estimated age of each animal recorded. The age was determined by analyzing the cementum lines of the canine teeth as part of a monitoring program [26, 27]. The eyes of the animals were examined for the presence of nematodes by lifting the nictitating membrane. The total number of parasites per animal was recorded; nematodes were collected and preserved in 96% ethanol for subsequent morphological and molecular analysis. Morphological identification of *T. callipaeda* was conducted following descriptions of Otranto et al. [28] and Otranto and Dutto [4]. The sex of *T. callipaeda* and the presence of larvae in female specimens were noted for each animal. For photographic documentation of the morphological characteristics, nematodes were mounted on glass slides, cleared with lactophenol, and photographed using Stereo Discovery v20 (Zeiss, Jena, Germany) and the microscope Imager M.2 (Zeiss), along with the software Axiovision and ZEN2 Pro [Blue Edition, version no. 3.5.093.00010 (Zeiss)].

### Molecular characterization

One nematode from each animal was placed in a 2-ml tube and frozen at − 21 °C for subsequent molecular analysis. DNA extraction was performed using the Blood and Tissue Kit (Qiagen, Hilden, Germany) with the QIAcube automated extraction system (Qiagen, Hilden, Germany). Identification of *T. callipaeda* was confirmed via conventional PCR assay followed by of a 650-bp fragment of the COI gene using forward primer 5′- HCO2198 TAAACTTCAGGGTGACCAAAAAATCA LCO1490- 3′ and reverse primer 5′- GGTCAACAAATCATAAAGATATTGG- 3′ [29]. PCR reactions were performed using a total volume of 20 µl, with 1 µl of isolated DNA. The amplified products were analyzed using capillary electrophoresis (QIAxcel System^®^, QIAGEN) with size markers ranging from 100 to 2500 base pairs (bp). The samples were purified using ExoSAP-IT^®^ (USB Corp., Cleveland, OH, USA) and then sequenced in both directions by Macrogen Inc. (The Netherlands). The sequences were assembled using SeqMan Pro software, edited using EditSeq from the Lasergene software (DNASTAR, Madison WI, USA), and compared to existing sequences using BLAST.

### Statistical analysis

To evaluate differences in *T. callipaeda* prevalence among positive animals based on age, sex, month of detection, and county, we performed a one-way ANOVA. If ANOVA assumptions were not met, the nonparametric Kruskal-Wallis test was used after testing the data for normality and homoscedasticity with the Shapiro-Wilk W test and Levane test, followed by a nonparametric test for a trend between ordered groups. A chi-square test was used to assess associations between infection presence and categorical variables, such as geographical region. Differences were considered significant if the *p* value was < 0.05. Statistical analyses were performed using Stata 13.1 (StataCopr. 2016. Stata Statistical Software: Release 13.1, College Station, TX).

## Results

**Fig. 1:**
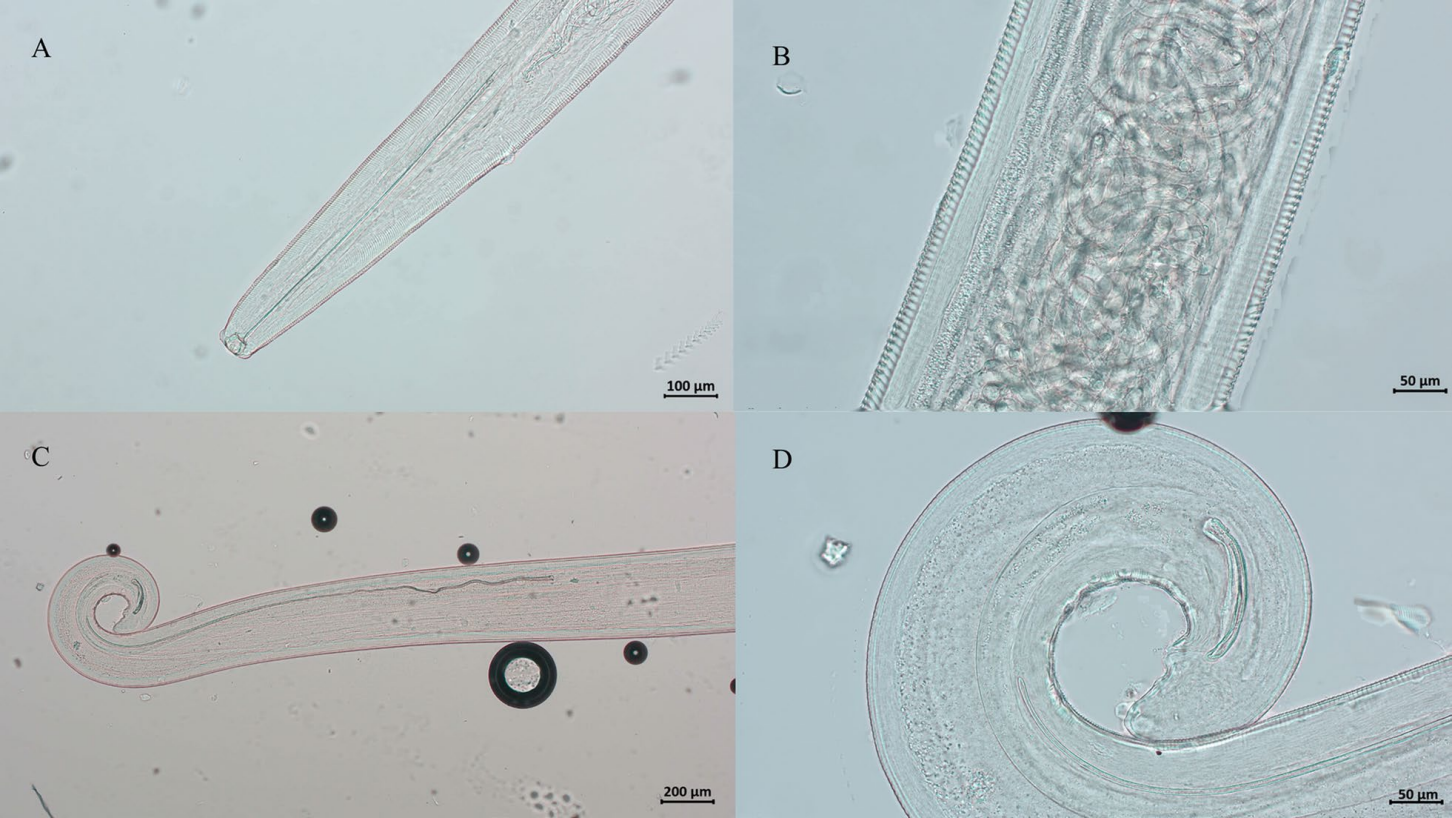
Morphological features of *Thelazia callipaeda* observed under light microscopy. (**A**) Anterior end of a female worm showing the buccal capsule. **B** Transverse striations along the cuticle and intrauterine larvae. **C** Coiled posterior end of a male worm with visible spicules. **D** Coiled posterior end of a female worm. Scale bars: 100 µm (**A**), 50 µm (**B** and **D**), 200 µm (**C**)

**Fig. 2:**
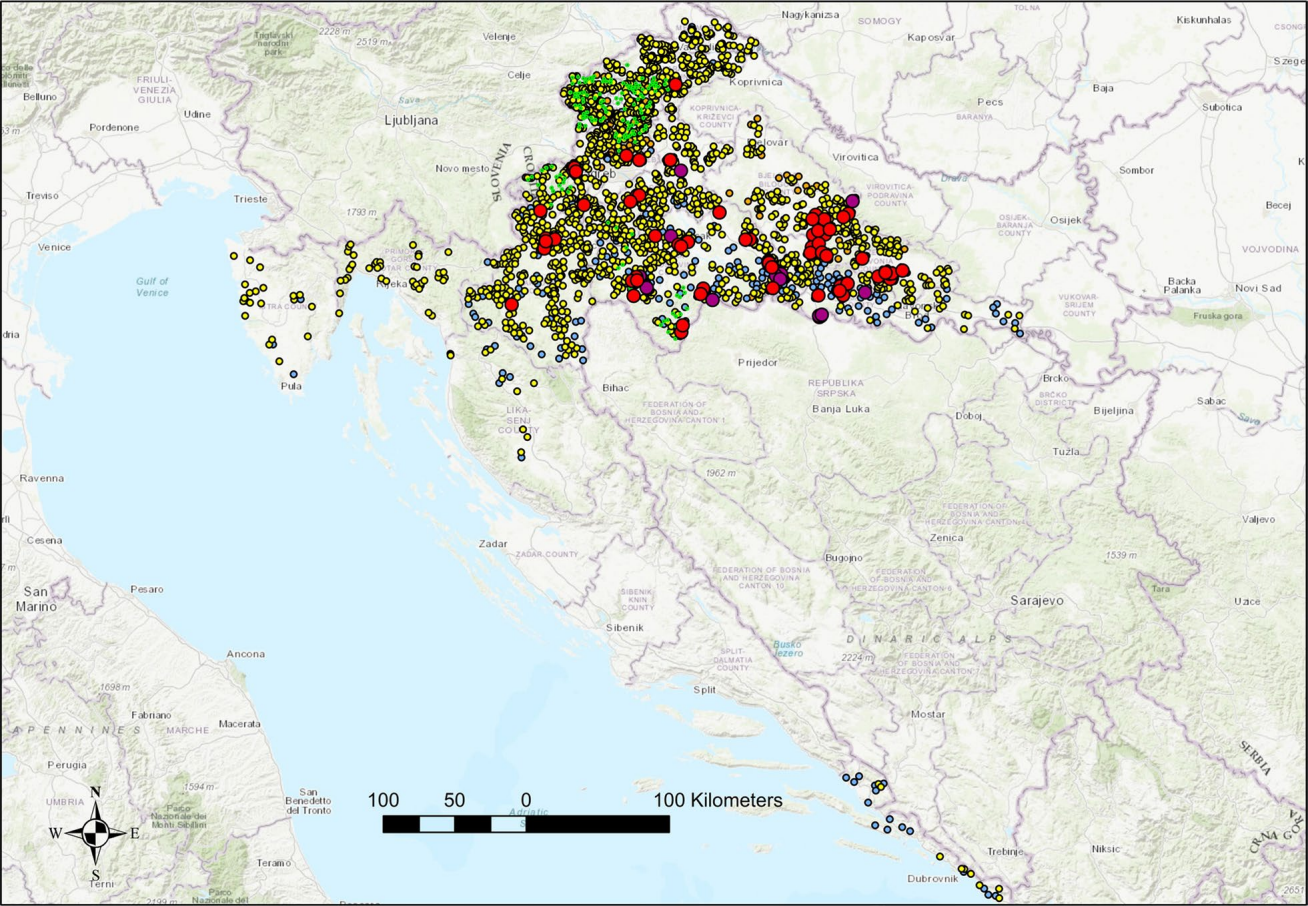
Geographic distribution of *Thelazia callipaeda* infections in wild carnivores across Croatia. Spatial distribution of examined red foxes, showing positive individuals (red circles) and negative individuals (green diamonds). Distribution of golden jackals, with positive cases marked by purple circles and negative cases by blue diamonds. Sampling locations for European badgers and stone martens, all of which tested negative. Brown diamonds indicate badger samples, while yellow diamonds represent pine martens

All parasites collected from red foxes and golden jackals were morphologically identified as *T. callipaeda* adults (Fig. 1). The overall prevalence was 3.9% (88/2,237; 95% CI 3.20–4.82), with red foxes showing a prevalence of 3.8% (69/1,794; 95% CI 3.00–4.84) and golden jackals 4.3% (19/443; 95% CI 2.40–6.18). All European badgers (0/180) and European pine martens (0/215) tested negative. There was no statistically significant difference in the prevalence between red foxes and golden jackals (*p* = 0.6677). Infected animals were found in eight continental counties but not in coastal counties of Primorje-Gorski Kotar and Lika Senj, Istra and Dubrovnik-Neretva, and in two continental counties Međimurje and Krapina-Zagorje (Fig. 2, Table 2).

**Table 2:** Prevalence of *Thelazia callipaeda* in red foxes and golden jackals by county in Croatia. This table presents the county-level prevalence of *T. callipaeda* in red foxes and golden jackals, including 95% confidence intervals (CIs) and statistical significance

County	Red fox			Golden jackal		
	Prevalence % (positive/examined)	95% CI	Significance	Prevalence % (positive/examined)	95% CI	Significance
Bjelovar-Bilogora County	9,9 (10/101)	4.85–17.46		16,7 (1/6)	0–59.51	
Požega-Slavonia County	10,7 (17/159)	6.35–16.57		0/2	0	
Brod-Posavina County	20.0 (7/35)	8.44–36.94		10.0 (7/70)	2.80–17.20	
Karlovac County	2,7 (7/264)	1.07–5.39		0/84	0	
Sisak-Moslavina County	6,6 (19/287)	4.03–10.15		5,5 (9/164)	1.97–9.01	
Primorje-Gorski Kotar County	0/41	0		0/1	0	
Lika-Senj County	0/10	0		0/6	0	
Istra County	0/20	0		0/4	0	
Dubrovnik-Neretva County	0/9	0		0/16	0	
The City of Zagreb	2.0 (1/50)	0.05–10.65		0/10	0	
Međimurje County	0/48	0		0/1	0	
Varaždin County	0,6 (1/181)	0.14–3.04		0/5	0	
Krapina-Zagorje County	0/265	0		0/4	0	
Zagreb County	2,2 (7/324)	0.87–4.40		2,9 (2/70)	0–6.86	
Total	3,8 (69/1794)	3.00–4.84	*p* = 0.001	4,3 (19/443)	2.39–6.18	*p* = 0.329

No positive cases were detected in several coastal and northwestern counties in either species

**Fig. 3:**
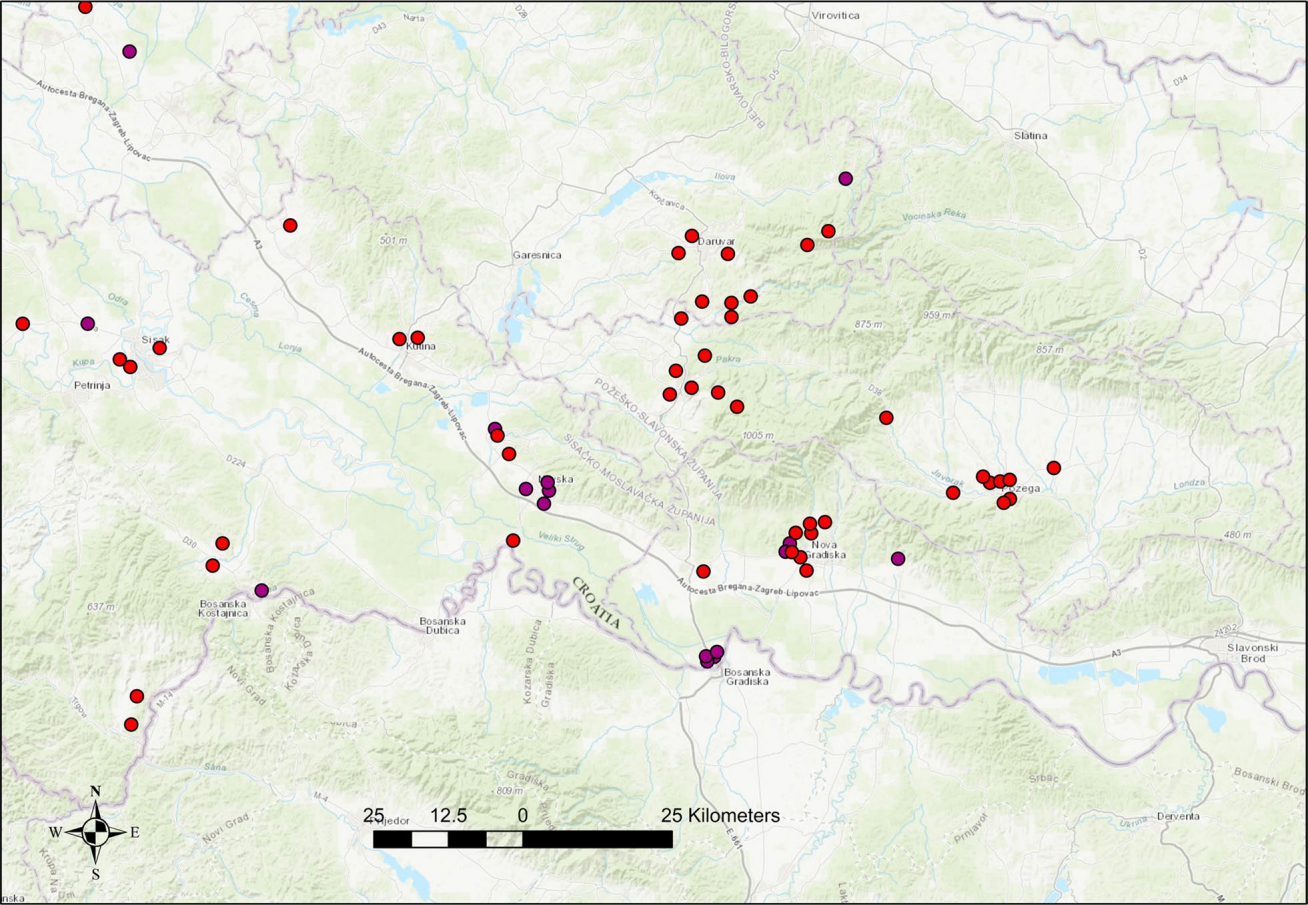
Spatial distribution of positive red foxes and golden jackals in the region with the highest infection density. The map shows the spatial clustering of wild carnivores infected with *Thelazia callipaeda* in central and eastern Croatia. Each red circle represents a confirmed positive red fox, while purple circles indicate positive golden jackals

**Fig. 4:**
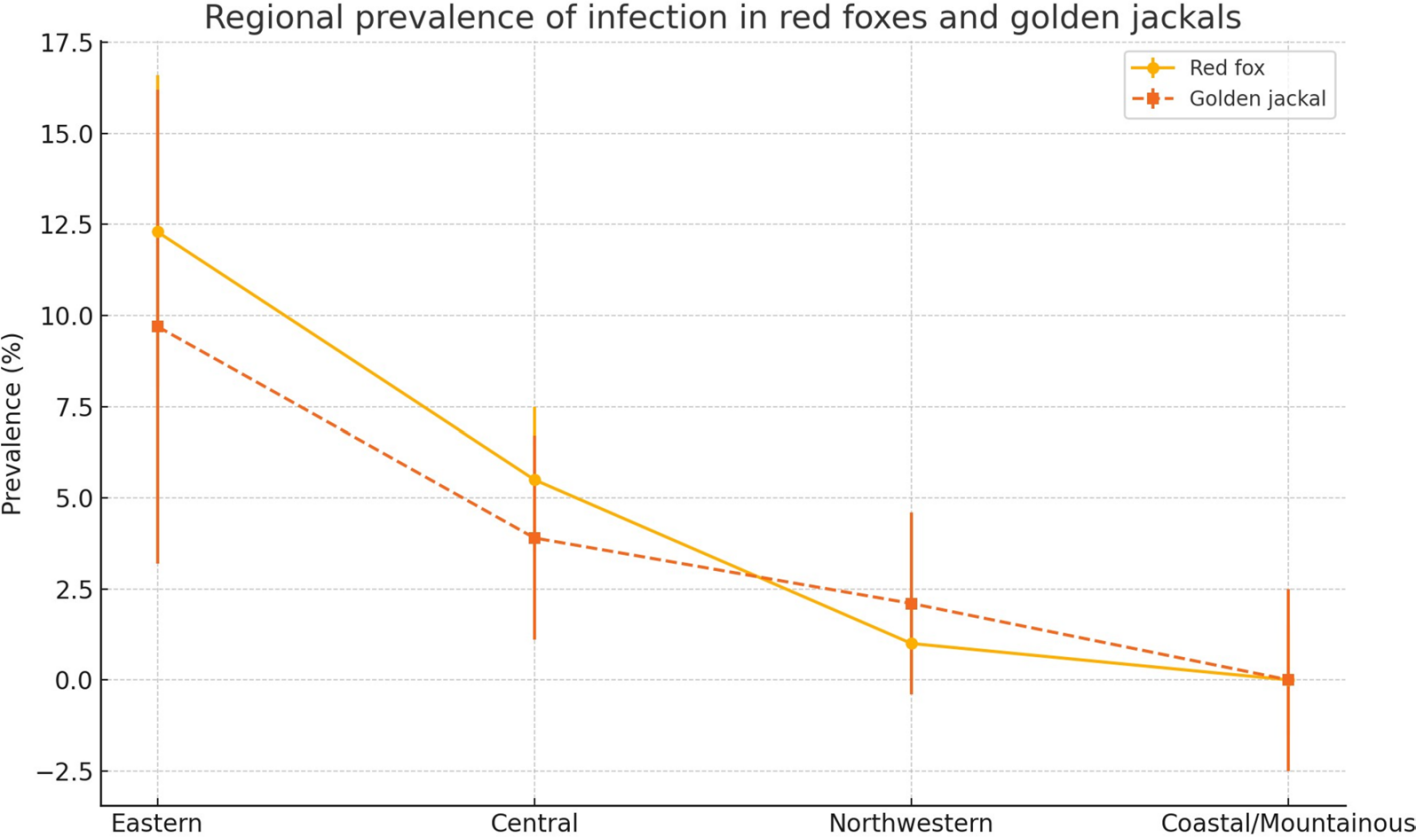
Regional prevalence of *Thelazia callipaeda* infection in red foxes and golden jackals across four major geographical regions of Croatia. The graph shows mean prevalence in eastern, central, northwestern, and coastal/mountainous regions. Red foxes exhibited significantly higher prevalence in Eastern Croatia, while no infections were detected in the coastal/mountainous region for either species

The overall prevalence in red foxes and golden jackals varied significantly across the four major geographical regions of Croatia. In red foxes, the highest prevalence was observed in Eastern Croatia (Slavonia) at 12.4% (24/194; 95% CI 8.05–17.91), followed by Central Croatia with 5.5% (36/652; 95% CI 3.90–7.52) and Northwestern Croatia with 1.0% (9/868; 95% CI 0.48–1.97) (Fig. 3). No positive cases were detected in the coastal and mountainous region (0/80; 95% CI 0–4.52). A chi-square test confirmed that these regional differences were highly significant (*p* < 0.0001). Among golden jackals, the highest prevalence was again recorded in Eastern Croatia at 9.7% (7/72; 95% CI 4.00–18.94), followed by Central Croatia (3.9% 10/254; 95% CI 1.91–7.11) and Northwestern Croatia (2.0%, 2/98; 95% CI 0.25–7.17). No positive animals were detected in the coastal and mountainous region (0/36; 95% CI 0–9.61). The differences in prevalence between regions were statistically significant (*p* = 0.039), although less pronounced than in red foxes (Fig. 4). When analyzing the prevalence in red foxes between counties, significant differences were found (*p* = 0.001), with the highest prevalence in Brod-Posavina County at 20.0%, followed by Požega-Slavonia County at 10.7%, Bjelovar-Bilogora County at 9.9%, and Sisak-Moslavina County at 6.6%. A prevalence of < 3.0% was found in Karlovac County (2.7%), 2.2% in Zagreb County, 2.0% in the City of Zagreb and 0.6% in Varaždin County. The infected golden jackals originated from the four counties namely Bjelovar-Bilogora, Brod-Posavina, Sisak-Moslavina, and Zagreb County, with a prevalence of 16.7%, 10.0%, 5.5%, and 2.9%, respectively (Table 2). Despite the different prevalences in the counties, which ranged from zero to 16.7%, the differences between the counties for the golden jackal were not significant (*p* = 0.329) (Table 2).

In red foxes, infection prevalence varied across age categories, peaking in 2–3-year-old animals and gradually declining with age. Although the highest prevalence in jackals was also observed in younger age groups (< 1 and 2–3 years), the wide confidence intervals, due to smaller sample sizes, limit interpretation. No infections were detected in jackals aged 5–6 and 6–7 years. The differences in prevalence by age were not statistically significant for either species (*p* = 0.066 for red foxes; *p* = 0.561 for golden jackals), although a trend toward higher prevalence in younger red foxes was noted (Table 3). There were no statistically significant differences by sex in either species (*p* = 0.479 for red foxes; *p* = 1.000 for jackals) (Table 3).

**Table 3:** Age- and sex-related prevalence of *Thelazia callipaeda* in red foxes and golden jackals

Age category (years)	Red fox			Golden jackal		
	Prevalence (positive/examined)	95% CI	Significance	Prevalence (positive/examined)	95% CI	Significance
< 1	1.3% (3/229)	0.27–3.78	*p* = 0.057	9.1% (2/22)	0.0–22.14	*p* = 0.998
1–2	4.4% (19/433)	2.66–6.77		5.1% (6/118)	1.06–9.11	
2–3	5.7% (26/455)	3.81–8.56		6.3% (6/95)	1.34–11.30	
3–4	4.2% (12/288)	2.17–7.17		3.7% (2/54)	0–8.91	
4–5	2.3% (3/133)	0.47–6.45		4.3% (2/46)	0–10.47	
5–6	4.2% (4/95)	1.16–10.43		0% (0/58)	0	
6–7	2.4% (1/42)	0.06–12.57		0% (0/13)	0	
7 >	0.8% (1/129)	0.02–4.24		2.7% (1/37)	0.02–10.47	
Female	4.22% (38/900)	3.00–5.75	*p* = 0,406	4.1% (8/196)	1.29–6.88	*p* = 0.848
Male	3.5% (31/894)	2.37–4.89		4.5% (11/247)	1.86–7.04	

This table summarizes the prevalence of *Thelazia callipaeda* infections across different age categories and between sexes in red foxes and golden jackals

**Fig. 5:**
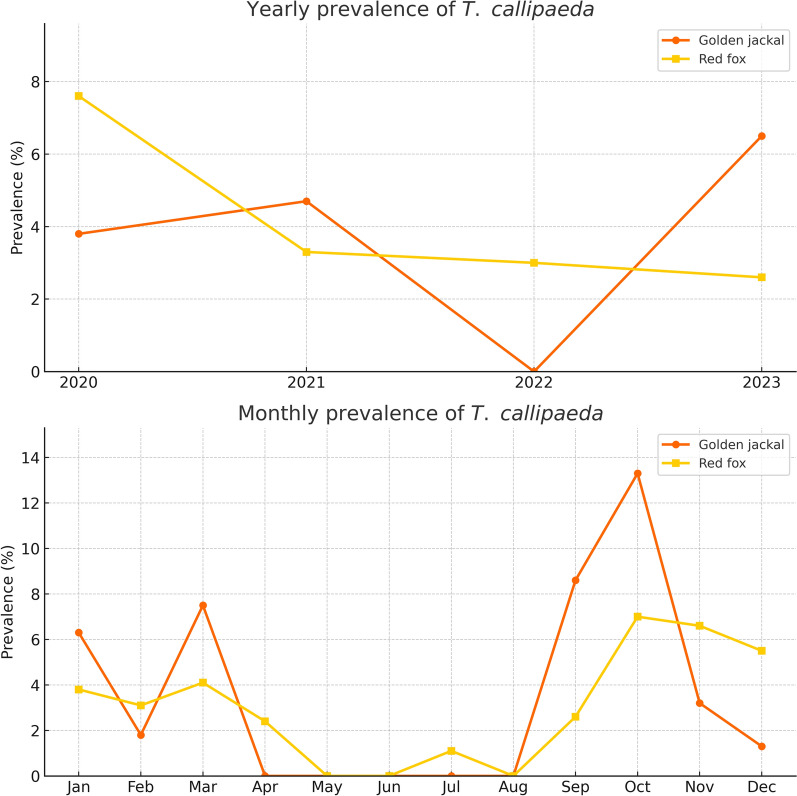
Yearly and monthly prevalence of *Thelazia callipaeda* infection in red foxes and golden jackals. Top panel: The yearly prevalence of infection from 2020 to 2023 demonstrates a significant declining trend in red foxes, while golden jackals show a fluctuating pattern with a notable increase in 2023. Bottom panel: Monthly prevalence data indicate clear seasonal variation in red foxes, with infection rates peaking during October–December. In golden jackals, infections were more sporadic but also highest in autumn months

A clear temporal trend in *T. callipaeda* prevalence was observed in red foxes, with a significant decline over the 4-year study period (*p* = 0.0008). The highest prevalence was recorded in 2020, followed by a progressive decrease through 2023, suggesting potential changes in exposure risk, vector abundance, or host–pathogen dynamics. In contrast, prevalence in golden jackals showed a non-significant increasing trend (*p* = 0.066), peaking in 2023, although no positive cases were detected in 2022 (Table 4, Fig. 5).

**Table 4:** Temporal distribution of *Thelazia callipaeda* infection in red foxes and golden jackals by year and month

Year	Red fox			Golden jackal		
	Prevalence % (positive/examined)	95% CI	Significance	Prevalence % (positive/examined)	95% CI	Significance
2020	7.6 (26/340)	5.55–12.35	*p* = 0.0008	3.8 (2/52)	0.97–16.44	*p* = 0.829
2021	3.3 (14/420)	2.02–5.87		4.7 (4/85)	1.81–13.48	
2022	3.0 (14/461)	1.84–5.33		0.0 (0/106)	0	
2023	2.6 (15/573)	1.61–4.49		6.5 (13/200)	3.96–12.20	

Month	Prevalence % (positive/ examined)	95% CI	Significance	Prevalence % (positive/ examined)	95% CI	Significance
Jan	3.8 (9/236)	1.76–7.12		6.3 (3/48)	0–13.353	
Feb	3.1 (8/262)	1.33–5.93		1.8 (1/56)	0–5.36	
Mar	4.1 (6/147)	1.51–8.67		7.5 (3/40)	0–16.03	
Apr	2.4 (1/41)	0.06–12.86		0 (0/9)	0	
May	0 (0/74)	0		0 (0/19)	0	
Jun	0 (0/96)	0		0 (0/24)	0	
Jul	1.1 (1/90)	0.02–6.04		0 (0/12)	0	
Aug	0 (0/88)	0		0 (0/16)	0	
Sep	2.6 (2/76)	0.03–9.18		8.6 (3/35)	0–18.33	
Oct	7.0 (9/129)	3.24–12.83		13.3 (6/45)	3.01–26.66	
Nov	6.6 (15/227)	3.75–10.66		3.2 (2/63)	0–7.63	
Dec	5.5 (18/328)	3.28–8.53		1.3 (1/76)	0–3.94	
Total	3.8 (69/1794)	3.00–4.84	*p* = 0.014	4.3 (19/443)	2.39–6.18	*p* = 0.997

This table presents annual and monthly prevalence trends of *Thelazia callipaeda* in red foxes and golden jackals from 2020 to 2023

Seasonal variation was also evident, particularly in red foxes, where infection prevalence differed significantly across months (*p* = 0.014). The highest rates were observed in autumn and early winter, with peaks in October, November, and December, and no positive cases during late spring and summer (May to August). In golden jackals, the highest monthly prevalence was also recorded in autumn, especially in October; however, the differences were not statistically significant (*p* = 0.076), likely because of smaller and uneven sample sizes. These findings indicate a marked seasonal pattern in foxes and a more variable trend in jackals, with transmission likely occurring in late summer and autumn, followed by overwintering of adult nematodes in the definitive hosts (Table 4, Fig. 5).

A total of 690 nematodes were recovered from 88 infected wild carnivores, comprising 557 parasites from red foxes and 133 from golden jackals. The sex ratio was skewed toward females in both species, with a male-to-female ratio of 1:2.0 in red foxes and 1:2.2 in golden jackals. Females carrying larvae were found in 56.5% of infested red foxes (39/69) and in 73.7% of infested jackals (14/19), suggesting potential differences in reproductive dynamics or host susceptibility. The intensity of infection ranged from one to 63 parasites per fox (mean = 8.1) and 1 to 40 per jackal (mean = 7.0). Although the mean parasite burden was slightly higher in red foxes, the overall intensity was comparable between species (Table 5).

**Table 5:** Distribution and reproductive status of *Thelazia callipaeda* nematodes by county in red foxes and golden jackals

County	Red fox				Golden jackal			
	Male	Female	Total	Foxes with female with larvae/positive foxes	Male	Female	Total	Golden jackals with female with larvae/positive golden jackals
Bjelovar-Bilogora County	15	33	48	80.0% (8/10)	4	6	10	100.0% (1/1)
Požega-Slavonia County	104	200	304	70.6% (12/17)	0	0	0	0
Brod-Posavina County	18	26	44	71.4% (5/7)	28	50	78	85.7% (6/7)
Karlovac County	9	18	27	28.6% (2/7)	0	0	0	0
Sisak-Moslavina County	31	82	113	47.4% (9/19)	8	32	40	66.7% (6/9)
Primorje-Gorski Kotar County	0	0	0	0	0	0	0	0
Lika-Senj County	0	0	0	0	0	0	0	0
Istra County	0	0	0	0	0	0	0	0
Dubrovnik-Neretva County	0	0	0	0	0	0	0	0
The City of Zagreb	1	1	2	100.0% (1/1)	0	0	0	0
Međimurje County	0	0	0	0	0	0	0	0
Varaždin County	0	3	3	0/1	0	0	0	0
Krapina-Zagorje County	0	0	0	0	0	0	0	0
Zagreb County	5	11	16	28.6% (2/7)	1	4	5	50.0% (1/2)
Total	183	374	557	56.5% (39/69)	41	92	133	73.7% (14/19)

This table presents the total number and sex of *Thelazia callipaeda* nematodes recovered per county, along with the proportion of infected animals harboring gravid females

 Parasitological analysis revealed quantitative and reproductive differences in *T. callipaeda* infection dynamics between red foxes and golden jackals across Croatian counties (Table 5). In red foxes, most infections occurred in Požega-Slavonia, Sisak-Moslavina, and Bjelovar-Bilogora, with 56.5% of infected animals carrying gravid females. Golden jackals had fewer parasites overall but a higher proportion of reproductively active infections (73.7%), especially in Bjelovar-Bilogora and Brod-Posavina counties. Correlation analysis showed a weak to moderate relationship between prevalence and both parasite load (*r* = 0.36) and number of gravid females (*r* = 0.56) in red foxes, whereas these associations were much stronger in golden jackals (*r* = 0.94 and *r* = 0.87), respectively.

**Fig. 6:**
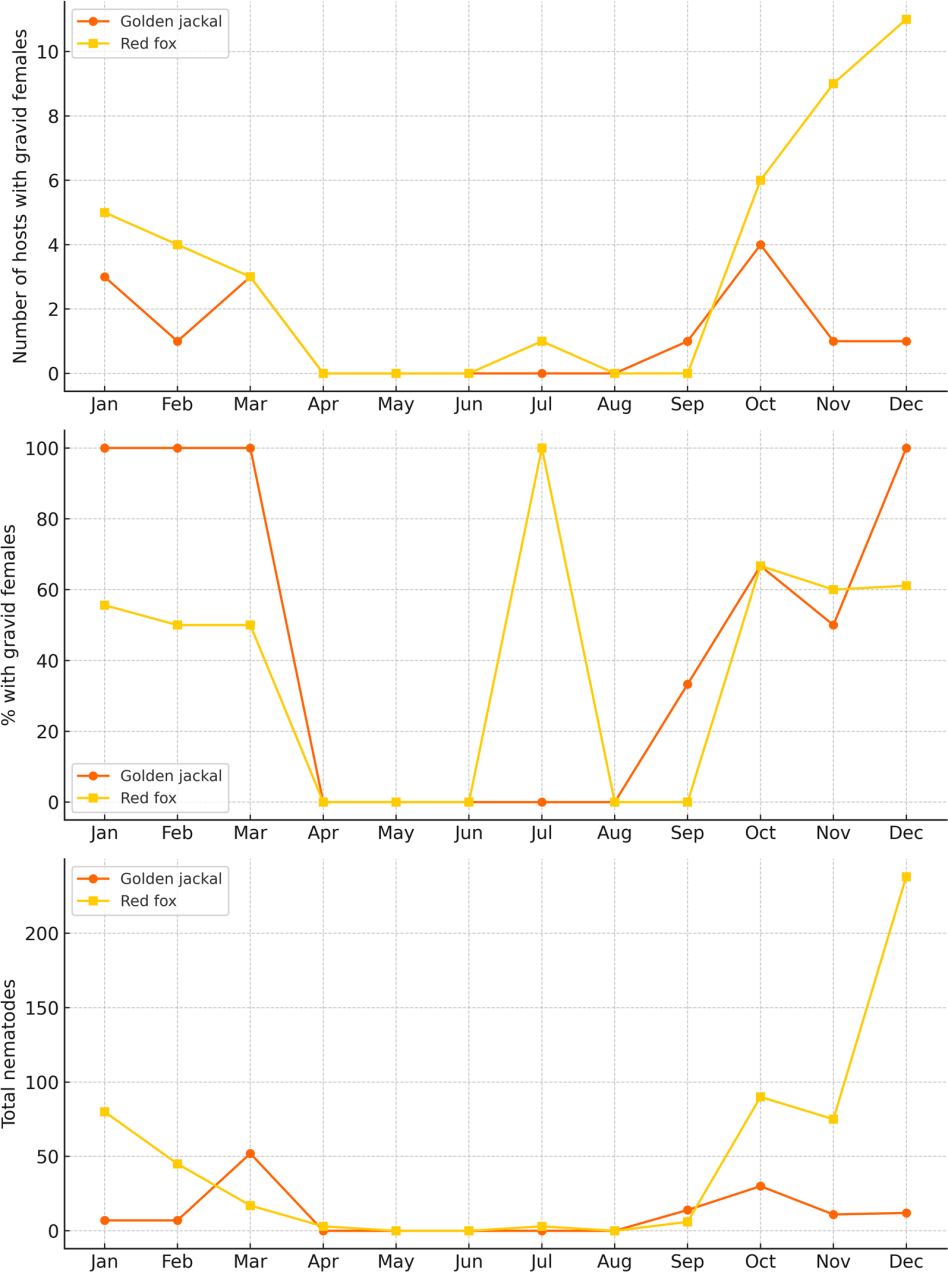
Monthly reproductive dynamics and parasite burden of *Thelazia callipaeda* in red foxes and golden jackals. Top panel: Number of infected hosts carrying gravid females by month. Red foxes exhibited a pronounced seasonal pattern, with the highest number of gravid infections observed in late autumn and winter (October–December). Middle panel: Percentage of infected hosts harboring gravid females. Golden jackals maintained high reproductive activity throughout the year when infections occurred, whereas red foxes showed marked fluctuations, with peak reproductive rates during the colder months. Bottom panel: Total number of *T. callipaeda* nematodes recovered monthly. The parasite burden in red foxes was markedly higher in winter months, especially in December, whereas in jackals, nematode numbers were lower and more stable, with a modest increase in autumn

Seasonal analysis revealed clear patterns in *T. callipaeda* infection dynamics. In red foxes, both parasite burden and the number of infections with gravid females peaked during winter and autumn, with minimal activity in spring and summer. In contrast, golden jackals showed the highest parasite counts in autumn and spring, while no infections were recorded during summer. Gravid females were most frequently detected in colder months in both hosts (Table 6, Fig. 6). These findings support a strong seasonal trend in red foxes, confirmed by a significant difference in total worm burden across seasons (Kruskal-Wallis test, *p* = 0.044), while no statistically significant seasonal variation was observed in golden jackals (*p* = 0.123).

**Table 6:** Seasonal distribution and reproductive activity of *Thelazia callipaeda* nematodes by month in red foxes and golden jackals

*T. callipaeda*	Red fox				Golden jackal			
Month	Male	Female	Total	Foxes with female with larvae/positive foxes	Male	Female	Total	Golden jackals female with larvae/positive golden jackals
Jan	28	52	80	55.6% (5/9)	1	6	7	100.0% (3/3)
Feb	12	33	45	50.0% (4/8)	0	7	7	100.0% (1/1)
Mar	3	14	17	50.0% (3/6)	17	35	52	100.0% (3/3)
Apr	0	3	3	0/1	0	0	0	0
May	0	0	0	0	0	0	0	0
Jun	0	0	0	0	0	0	0	0
Jul	0	3	3	100.0% (1/1)	0	0	0	0
Aug	0	0	0	0	0	0	0	0
Sep	2	4	6	0/2	2	12	14	33.3% (1/3)
Oct	26	64	90	66.7% (6/9)	11	19	30	66.7% (4/6)
Nov	21	54	75	60.0% (9/15)	3	8	11	50.0% (1/2)
Dec	91	147	238	61.1% (11/18)	7	5	12	100.0% (1/1)
Total	183	374	557	56.5% (39/69)	41	92	133	73.7% (14/19)

This table details the monthly distribution of *Thelazia callipaeda* nematodes by sex, along with the proportion of infected hosts harboring gravid females

Molecular analysis confirmed that all 88 nematodes examined belong to haplotype 1, the sole haplotype of *T. callipaeda* present in Europe. A representative sequence was deposited in the GenBank database under accession no. PV550083.

## Discussion

This study represents the first comprehensive investigation of *T. callipaeda* in wild carnivores in Croatia, providing key insights into its prevalence, spatial distribution, and reproductive ecology in two major host species, red foxes and golden jackals.

The overall prevalence in wild canids in Croatia was low (3.9%), aligning with reports from other parts of Central Europe, such as Switzerland (5.6%) [21] and Slovakia (1.3%) [20]. However, it was lower than the prevalence recorded in southeastern Europe, including Bosnia and Herzegovina (27.7%) [22] and Romania (29.4%) [23]. Higher prevalence in Eastern and Central Croatia, particularly in Požeško-slavonska and Brodsko-posavska counties, suggests the presence of localized transmission hotspots, likely driven by favorable environmental conditions for both vectors and hosts. In contrast, the absence of infection in the coastal and mountainous areas may reflect unsuitable habitats for *P. variegata* or reduced host-vector interactions [22, 30].

Although red foxes and golden jackals exhibited similar overall prevalence, their infection dynamics differed significantly. In foxes, a more pronounced temporal variation and clear seasonal pattern were observed, with infection rates peaking during the colder months. This seasonality was further supported by significant differences in monthly prevalence and parasite burden, suggesting increased exposure and transmission during autumn and winter [13, 16]. In contrast, golden jackals exhibited a more stable pattern of infection throughout the year, suggesting a potential role in maintaining low-level transmission year-round. The presence of adult *T. callipaeda* nematodes in foxes and jackals during late winter and early spring may represent overwintering individuals, as environmental conditions during this period are suboptimal for vector activity. This observation supports the hypothesis that infections acquired in autumn and early winter can persist for several months, contributing to early in the year parasite detection even in the absence of active transmission [13, 16].

Female nematodes predominated in both host species, with a high proportion of infections involving gravid females, especially in jackals (73.7%). Notably, female-only infections were significantly more frequent than male-only infections in both species, with red foxes showing a particularly high proportion of infections involving only females (40.6%). These findings could reflect longer female worm longevity or sex-biased survival, as hypothesized in previous studies [13, 16, 19, 21].

Although red foxes harbored more nematodes and had a slightly higher mean burden, golden jackals showed stronger correlations among prevalence, total worm counts, and the presence of gravid females. This suggests that jackals may play a more stable and reproductively effective role in the epidemiology of *T. callipaeda*, particularly in areas with persistent transmission [8].

The mean intensity of infection in red foxes (8.1 nematodes per animal) closely matches data from Bosnia and Herzegovina (8.1) [22] and Portugal (8.5) [31] and falls between the lower and upper extremes reported across Europe, 3.8 in Switzerland [21] to 23.2 in northern Romania [23], indicating moderate transmission pressure in Croatia.

Temporal dynamics provide further support for the hypothesis that red foxes may act as reservoir hosts during peak transmission seasons, while golden jackals potentially function as reservoirs across extended periods. This complementary dynamic likely facilitates the persistence of *T. callipaeda* in wild carnivore populations and may contribute to zoonotic spillover risk in endemic areas [7].

Few studies have investigated *T. callipaeda* infections in European badgers and stone martens across Europe. Despite the present study including the largest examined sample to date (180 European badgers and 215 stone martens), no infections were detected. This contrasts with isolated reports from Italy, Portugal, and Romania [7, 24, 32].

The absence of infection in badgers and martens may be explained by their predominantly nocturnal activity and limited spatial–temporal overlap with the diurnal vector *P. variegata*, resulting in reduced host-vector contact and thus a lower risk of transmission [7, 33]. Moreover, high prevalence in primary host species, such as red foxes, may be needed for spillover to occur. This is supported by spatial distribution data: only one infected fox was detected in northwestern Croatia, the region with the highest density of sampled badgers and martens, while the areas with the highest prevalence of *T. callipaeda* in foxes and golden jackals had the lowest number of sampled badgers and martens.

Findings align with those from neighboring countries. For example, studies from Romania and Bosnia and Herzegovina reported similarly high prevalence and reproductive activity in red foxes, with comparable seasonality and geographical clustering [22, 23]. In contrast, lower prevalence has been observed in Switzerland and Slovakia, likely reflecting climatic constraints on vector activity. Multi-host surveillance, as used in recent Italian and Romanian studies, has proven effective in capturing broader epidemiological trends and can inform public health preparedness in regions with overlapping wildlife, domestic animals, and human populations [10, 14].

Taken together, these results highlight the importance of multi-host surveillance in understanding the eco-epidemiology of *T. callipaeda*. The integration of molecular diagnostics, geographical mapping, and host-parasite ecology offers a robust framework for monitoring this emerging zoonotic parasite in southeastern Europe. Further studies should explore the influence of vector dynamics and climate on seasonal transmission and assess the risk of cross-species transmission to domestic animals and humans.

## Conclusions

This study provides the first systematic evidence of *T. callipaeda* infection in wild carnivores in Croatia, highlightening significant differences in prevalence, seasonal dynamics, and reproductive indicators between red foxes and golden jackals. The spatial and temporal heterogeneity in parasite distribution, along with the predominance of female and gravid nematodes, underscores the complex eco-epidemiological patterns of this zoonotic parasite. Red foxes appear to serve as seasonal reservoirs of infection during colder months, while golden jackals may act as stable reservoirs, maintaining low-level transmission throughout the year. The absence of infection in other sympatric carnivores such as badgers and martens emphasizes the importance of host ecology and vector contact in shaping transmission risk. These findings support the need for continued surveillance and integrated monitoring of *T. callipaeda* in wildlife populations to better understand its transmission ecology and assess zoonotic potential across Europe.

## Data Availability

Data supporting the main conclusions of this study are included in the manuscript.
